# Survivin: a unique target for tumor therapy

**DOI:** 10.1186/s12935-016-0326-1

**Published:** 2016-06-23

**Authors:** Himani Garg, Prerna Suri, Jagdish C. Gupta, G. P. Talwar, Shweta Dubey

**Affiliations:** Amity Institute of Virology and Immunology, Amity University Uttar Pradesh, J-3 Block, Room No: LG21, Sector 125, Noida, Uttar Pradesh 201303 India; Amity Institute of Biotechnology, Amity University Uttar Pradesh, Sector 125, Noida, India; Talwar Research Foundation, E-8 Neb Valley, Neb Sarai, New Delhi, 110 068 India

**Keywords:** Survivin, Immunotherapy, Apoptosis, IAPs

## Abstract

Survivin is the smallest member of the Inhibitor of apoptosis (IAP) family of proteins, involved in inhibition of apoptosis and regulation of cell cycle. These functional attributes make Survivin a unique protein exhibiting divergent functions i.e. regulating cell proliferation and cell death. Expression pattern of Survivin is also distinctive; it is prominently expressed during embryonal development, absent in most normal, terminally differentiated tissues but upregulated in a variety of human cancers. Expression of Survivin in tumours correlates with not only inhibition of apoptosis and a decreased rate of cell death, but also resistance to chemotherapy and aggressiveness of tumours. Therefore, Survivin is an important target for cancer vaccines and therapeutics. Survivin has also been found to be prominently expressed on both human and embryonic stem cells and many somatic stem cell types indicating its yet unexplored role in stem cell generation and maintenance. Overall, Survivin emerges as a molecule with much wider role in cellular homeostasis. This review will discuss various aspects of Survivin biology and its role in regulation of apoptosis, cell division, chemo-resistance and tumour progression. Various molecular and immunotherapeutic approaches targeting Survivin will also be discussed.

## Background

Cancer is a heterogeneous group of diseases where abnormal cell growth with potential to invade other body parts takes control of normal homeostasis and becomes fatal if not timely and rightly treated. While standard treatments like surgery, chemotherapy or radiotherapy have significantly improved the disease outcome, occurrence of drug resistance and metastatic spread of the disease still remains a tough challenge. Substantial data suggests that immunotherapy could serve as a powerful weapon to prevent metastatic spread of cancer. Immunotherapy specifically targets tumor cells thereby avoiding collateral damage to non-tumor cells. Induction of anti-tumor response also has the potential to eradicate tumor at distant sites in the body which may not be possible by surgical resection. Induction or enhancement of anti-tumor immune response is a formidable challenge in cancer because tumor cells use multiple evasion strategies and avoid being detected or eliminated by immune cells. Resistance to apoptosis is one important evasion mechanism by which tumour cells escape detection by immune cells and promote their proliferation at the same time. Therefore, molecules involved in regulation of apoptosis can be potential targets for tumor therapy including immunotherapy.

Inhibitor of apoptosis protein family (IAPs) is an important group of proteins involved in regulation of apoptosis. IAPs also have an important role in regulation of T cell responses in anti-tumor immunity. One member of this protein family, Survivin occupies a key position because of overexpression in cancer cells. It is speculated that Survivin overexpression in tumor cells promotes tumor progression by multiple pathways such as dysregulation of apoptosis and cell division, altered sensitivity to antitumor drugs or promoting survival of cancer stem cells. Survivin can serve as a universal tumor antigen because it is expressed in most human malignancies and has the potential to trigger immune effector responses. Therefore, blocking Survivin function by various immunotherapeutic or molecular approaches is emerging as a promising therapeutic strategy in cancer. This review will discuss various aspects of Survivin biology and multiple approaches to block Survivin in tumor cells.

### IAP family and Survivin

Inhibitors of apoptosis (IAP) family of proteins are found in almost all species from lower to higher vertebrates. Initially identified in baculoviruses as apoptotic suppressors, eight IAP homologs namely neuronal apoptosis inhibitory protein (NAIP), baculoviral IAP repeat-containing protein 2/human inhibitor of apoptosis protein-2 (c-IAP1/HIAP-2), baculoviral IAP repeat-containing protein3/human inhibitor of apoptosis protein-1 (c-IAP2/HIAP-1), X-linked inhibitor of apoptosis (XIAP), IAP-like protein 2 (ILP2), melanoma inhibitor of apoptosis protein (MLIAP), Survivin, and BIR repeat-containing ubiquitin enzyme system (BRUCE) have been identified in humans till date [1–10]. IAPs are functionally and structurally similar and function in regulation of programmed cell death [11–13]. Since IAPs are an important protein family which regulate cell fate in response to stress signals or genomic instability, therefore any dysregulation in IAP function has an obvious association with cancer development, induction of oncogenesis or drug resistance [14]. Investigating the mechanism of action of IAPs in cancer has provided important leads for anticancer drug development.

Structurally, all members of IAPs contain approximately 70 amino acid long Baculovirus IAP Repeats (BIR) domains at the N-terminus, which are essential but not sufficient for their anti-apoptotic activity [15]. Although the number of BIR domains varies among IAP members, each BIR domain is made up of cysteine and histidine residues in a well-defined pattern (Cx2Cx6Wx3Dx5Hx6C), which represents a novel zinc-binding fold. In addition to BIR domain, several viral, mammalian and insect IAPs require a ring finger domain (RING) near the C-terminus for suppression of apoptosis [9, 16]. However, C-terminal RING is not indispensable for suppression of apoptosis by human c-IAP1, c-IAP2 and XIAP [17, 18]. Human cIAP-1 and cIAP-2 also contain a Caspase Recruitment Domain (CARD), significance of which is not clearly understood during apoptosis suppression. IAPs also possess additional domains; ubiquitin-associated (UBA) domain (in c-IAP1, c-IAP2, XIAP and hILP2) and ubiquitin-conjugating (UBC) domain (in BRUCE/APOLLON), these domains assist in inducing ubiquitination and proteasome degradation of specific caspases and suppression of apoptosis [6, 19, 20]. Of all the IAPs known so far, Survivin is the smallest IAP protein with a single N-terminus BIR domain and C-terminus Coiled Coil (CC) domain. As compared to other IAPs, Survivin exhibits most restricted expression in adult tissues and has crucial role in regulating both cell division and apoptosis. It is highly expressed in most human cancers but not in normal, terminally differentiated adult tissues, thus making Survivin an exciting new tumour marker [5].

### Molecular organization and structure of Survivin

**Fig. 1:**
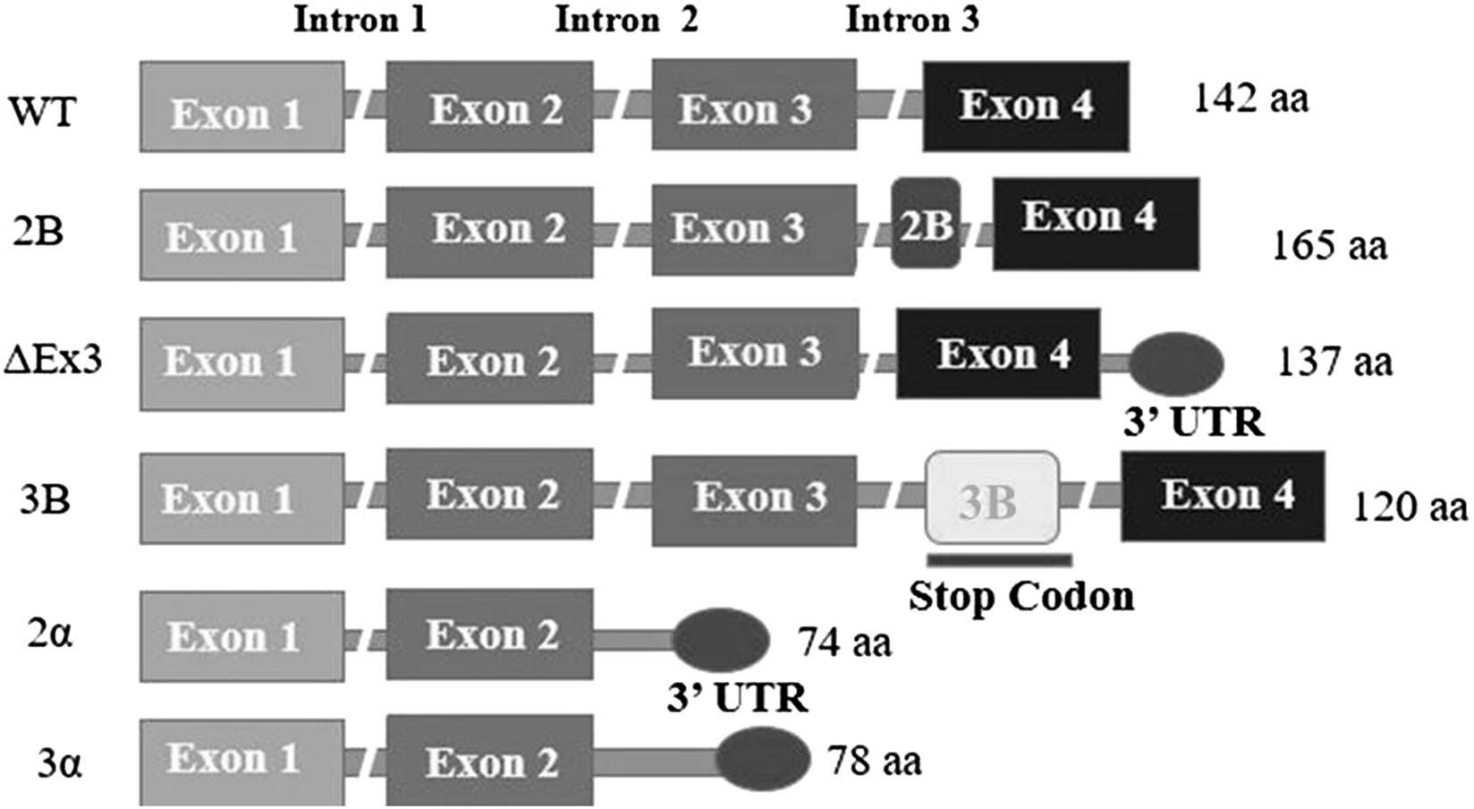
Alternative splice variants of Survivin encoded by BIRC5 gene. Schematic representation of exons encoding five isoforms of Survivin. Note the *breaks* in the* middle* denote introns 1–3. Wild type (WT) is coded by exons 1–4 and is 142 amino acid (aa) long. An additional 69 bp exon between exons 2 and 3 results in Survivin 2B, making it a total of 67 aa in length. Exon 3 deletion leads to a frame shift variant resulting in a 3′ UTR, forming Survivin ΔEx3, 137 aa in length. Survivin 3B is formed by addition of 7 aa at the C-terminal, exon 3B from intron 3, the first 113 aa are conserved from WT Survivin. Variants 2α and 3α have a 3′UTR along with exons 1–2 and are 74 and 78 aa in length respectively

Survivin is encoded by *BIRC5* gene and consists of 4 exons and 3 introns covering 14,796 nucleotides on chromosome 17q25 forming transcripts with varied functional domains. The *BIRC5* gene encodes wild type Survivin (WT, four exons; 142 amino acid) and five known additional splice variants i.e.; ΔEx3 (Survivin with deletion of exon 3; 137 amino acid), 2B (Survivin with an additional exon; 165 amino acids), 3B (five exons; 120 amino acid), 2α (2 exons;74 amino acids), 3α (two exons;78 amino acids) [21–23]. All Survivin isoforms share complete sequence identity in the N-terminus region, including some or the entire BIR domain, but they differ in the carboxyl end [24]. Figure 1 illustrates the various splice variants of Survivin and the amino acid alterations present in each splice variant. Survivin isoforms also have different expression patterns and cellular localization as compared to wild type form, Survivin-ΔEx3 is found predominantly in the nucleus where as Survivin- 2B is found in the cytoplasm. Alternative splicing of Survivin has been shown to have correlation with disease activity in various patient studies. Survivin WT, 2B and ΔEx3 variants have been extensively investigated for clinical and prognostic association in cancer. Presence of ΔEx3 variant has been associated with unfavourable clinical outcome and prognosis [25]. Conflicting data exists for clinical and pathological correlation of variant 2B in cancer; certain studies demonstrate association of 2B variant with aggravated disease and poor survival [26] while some studies indicate that presence of 2B variant is associated with less severe disease [27]. Overall, there is a consensus that ΔEx3 is anti-apoptotic and 2B is pro-apoptotic and that these variants may perform contrasting functions in tumor progression and response to therapy [28]. Presence of Survivin isoforms has also been shown to influence angiogenesis. In a study by Doucette T et al., presence of Survivin splice variant 2 was associated with poorer survival and promoted malignant progression, angiogenesis, and shorter tumor-free survival in mouse model of glioma [29].

It still remains unclear whether alternative splicing of Survivin is an adaptation used by cancer cells to support their proliferation and avoid detection by immune surveillance Association of splice variants with distinct pathological and survival outcomes indicate possible role of these variants in disease progression. However, relative contribution of different splice variants of Survivin with tumorigenesis, immune evasion and response to therapy is not completely understood and warrants further investigation.

### Cellular localization of Survivin

Survivin is predominantly present in the cytosol of tumour cells. However, a smaller nuclear fraction of Survivin localizing to kinetochores of metaphase chromosomes has also been reported in tumour and proliferating cells, indicating that these different subcellular pools of Survivin may have different functions [30, 31]. Cytosolic Survivin is believed to function as apoptotic suppressor while nuclear Survivin is postulated to regulate cell division. However, the pathological significance of nuclear Survivin as a favourable prognostic marker for tumour cells is still debatable. There is equivocal data from patient studies to indicate nuclear/cytosolic Survivin expression as an unfavourable or favourable prognostic marker in cancer [31]. Apart from cytosolic and nuclear pool, Survivin has also been detected in mitochondrion [32] and shown to be released into cytosol in response to cellular stress stimuli and suppress caspase activation [32]. Extracellular pool of Survivin has also been shown to exist as exosomes in form of 40–100 nm membrane vesicles secreted from tumour cells and taken up by surrounding cells [33]. In fact, many IAPs such as cIAP1, cIAP2 and XIAP including Survivin have been shown to exist as tumour exosomes in cancer cell lines [34]. Exosomes containing Survivin have been shown to re-enter cells and promote tumour growth. Khan et al. have demonstrated that extracellular pool of Survivin has the ability to cause neighbouring cancer cells to increase resistance to therapy, rapidly proliferate, and acquire an increased potential to become invasive in vitro [35, 36]. Increased levels of plasma derived exosomal Survivin from prostate cancer patients has been shown to correlate with disease severity [37]. Thus, both intracellular and extracellular release of Survivin in cancers may be responsible for aggravated disease.

## Multiple roles of Survivin

### Survivin and cell proliferation

Regulation of cell division is recognized as prominent function of Survivin. Normal cells show cell cycle dependent synthesis, expression and degradation of Survivin. Survivin forms an integral component of chromosomal passenger complex (CPC) which ensures proper segregation of chromosomes and cytokinesis during cell division [38]. Various checkpoints ensure nuclear division, attachment to mitotic spindle and cytokinesis. CPC is a hetero-tetrameric complex which localizes to different sites at different times during mitosis, this serves to regulate key events in cell division such as chromosome-microtubule attachment, proper spindle assembly and occurrence of cytokinesis. Aurora B kinase is the enzymatic component of CPC whereas as the other three components; inner centromere protein (INCENP), Survivin and Borealin (also known as Dasra) have regulatory and targeting functions. Alteration in any of the four components can lead to a defect in chromosomal segregation and/or cytokinesis and cause genomic instability [39–42]. Analysis of individual contributions of these proteins to formation of CPC suggests that the enzymatic component Aurora B kinase is directed to mitotic cell by the other three proteins of CPC i.e. INCENP, Survivin and Borealin/Dasra. INCENP acts as a scaffold protein and stabilizes the complex, Borealin acts to promote binding of Survivin to INCENP and Survivin acts as a determining factor in centromere localization of CPC [43, 44]. Although Survivin acts as a key protein in mediating CPC targeting, other protein components of CPC act to promote a stable structure. Furthermore, a distinct pool of subcellular Survivin is associated with polymerized tubulin and regulates microtubule formation during cell division.

### Survivin as an inhibitor of apoptosis

Overexpression of Survivin inhibits both intrinsic and extrinsic pathways of apoptosis [5, 45–48]. Depletion of Survivin in human cells induces defects in apoptosis and multiple defects in cell division [38, 49]. Although the mechanism of inhibition of apoptosis by Survivin is still unknown, both direct and indirect binding of Survivin to initiator or effector caspases is supposed to contribute to inhibition of apoptosis by Survivin [50]. Direct binding of Survivin to effector caspase-3 has been speculated, however, unlike other IAPs, Survivin does not possess the structural moiety responsible for docking of caspase-3 to the BIR domain [51]. Some studies suggest that Survivin binds directly to caspase-9 and inhibits its activity [52]. Another mechanism proposed for Survivin mediated inhibition of caspase- 9 is binding of hepatitis B X-interaction protein (HBXIP) to procaspase-9. It is also speculated that X-linked IAP (XIAP) which also contains BIR domain leads to an inhibition of caspase- 9 along with Survivin [53, 54]. In yet another mechanism, Survivin inhibits intrinsic (mitochondrial) pathway of apoptosis by binding to pro-apoptotic proteins called Secondary Mitochondria-derived Activator of Caspase (SMAC/DIABLO). SMAC/DIABLO are released during intrinsic pathway from mitochondria and activate caspase-9 to induce apoptosis. Survivin binds to SMAC/DIABLO and prevents caspase activation [52]. Song et al. have demonstrated that Survivin physically associates with SMAC/DIABLO and blocking this interaction induced apoptosis in taxol treated HeLa cells [55].

**Fig. 2:**
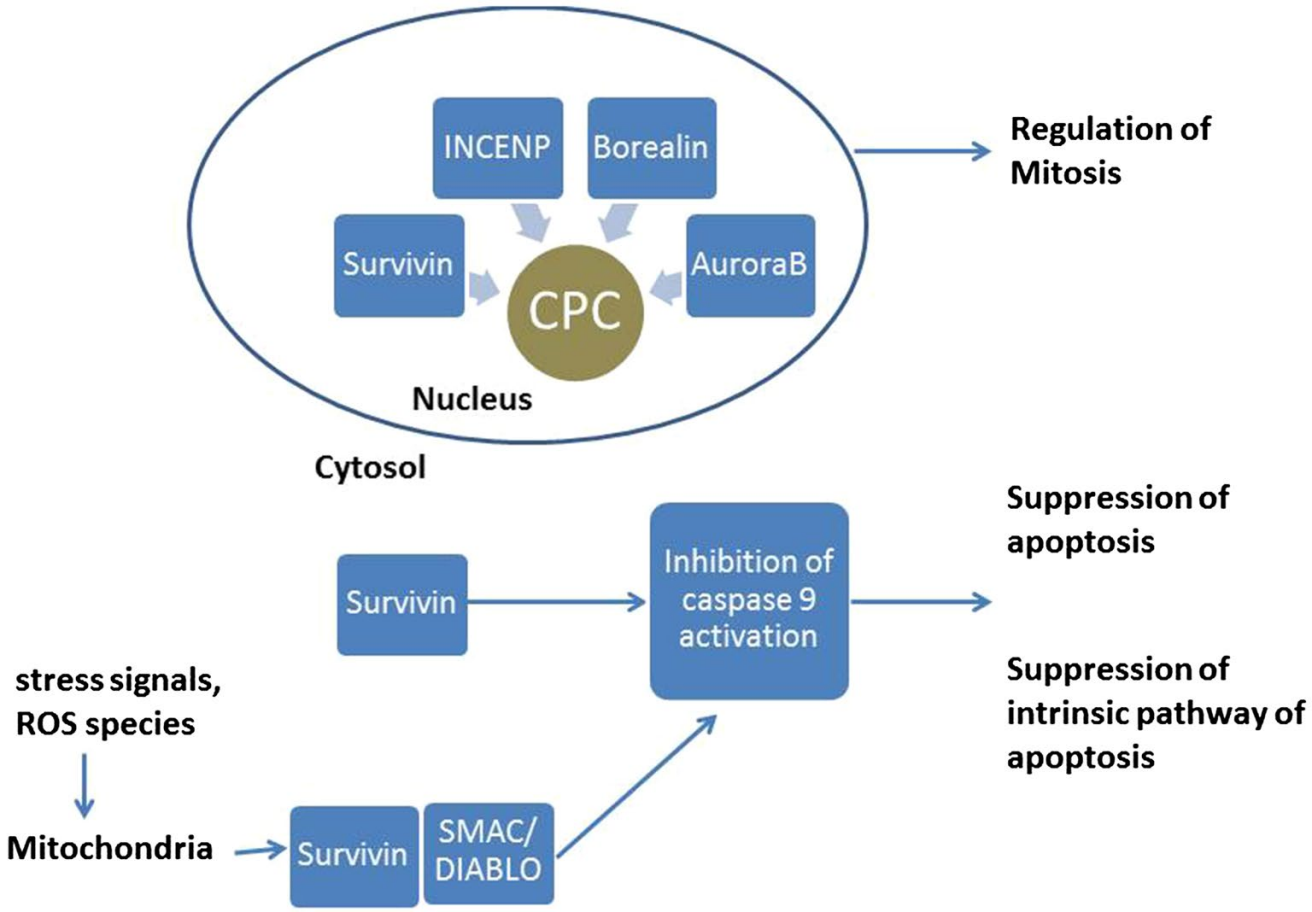
Unique role of Survivin in both apoptosis and cell division. Survivin expression in cytosol or mitochondrion can suppress both extrinsic and intrinsic pathways of apoptosis. Survivin expression in nucleus plays an important role in regulation of cell division by acting as an important component of chromosome passenger complex (CPC)

Taken together, there is substantial evidence to support the dual function of Survivin i.e. as a regulator of cell division and inhibitor of apoptosis (Fig. 2). Wheatley SP reported that the C-terminus of Survivin is required for cell division and the N- terminus is dispensable for apoptosis [56]. Though, thought as two separate functions, it may be possible that Survivin expression acts as a vital checkpoint for induction of programmed cell death in those cells undergoing aberrant cell division. More studies will be required to confirm whether Survivin exclusively acts as a regulator of cytokinesis or cell death in normal cells and whether either of these functions becomes predominant during tumor progression.

### Role of Survivin in supporting angiogenesis, metastasis and chemo-resistance of tumor cells

One of the pathways responsible for Survivin mediated tumor progression is promotion of angiogenesis in cancer cells. Survivin upregulates VEGF expression and promotes endothelial cell (ECs) proliferation by mechanisms which are still not clear [57]. Existence of a positive feedback loop connecting Survivin expression in tumor cells to PI3K/Akt enhanced β-catenin-Tcf/Lef-dependent transcription inducing secretion of VEGF and angiogenesis has been suggested [57]. Knockdown of Survivin in glioma has been shown to inhibit angiogenesis [58]. Small interfering RNA (siRNA) mediated silencing of Survivin sensitized human breast cancer cells to apoptosis and inhibited tumour formation and angiogenesis in breast or cervical cancer xenograft model in vivo [59]. Survivin induced VEGF expression also contributes to chemo-resistance by stimulating organization of tubulin into distinct fibres [60]. Survivin is particularly upregulated on tumor vascular ECs as compared to normal tissues, thus conferring drug resistance on tumor vascular ECs [61]. Therefore, targeting Survivin in tumor will promote not only tumor cell death but also sensitize cells of tumor vascular network to chemotherapeutic drugs.

Survivin may also cooperate with other IAP members to promote metastasis. Survivin overexpression enhanced migration of human melanocytes and melanoma cells on fibronectin whereas Survivin knockdown under sub-apoptotic conditions blocked their migration and invasion [62]. Inter-molecular interaction between XIAP and Survivin promoted tumor cell invasion in vitro and metastatic dissemination in vivo in murine model of breast cancer and rat insulinoma. This pathway operated independent of the role of IAPs in cell survival. Signal transduction through this pathway resulted in NF-κB activation, transcriptional upregulation of fibronectin, autocrine/paracrine signalling by β1 integrins, and constitutive phosphorylation, i.e. activation of cell motility kinases i.e.; FAK and Src. implicating direct involvement of IAPs in promoting metastasis. Most significantly, signal transduction via this pathway did not induce the traditional epithelial-mesenchymal transition (EMT) but rather induced an adhesion gene signature and many fold increase in expression of fibronectin gene in tumor cells [63]. Survivin has also been shown to enhance melanoma cell metastasis through integrin upregulation [64]. Survivin promoted breast cancer lymphatic metastasis through cooperation with vascular endothelial growth factor-C (VEGF-C) [65]. Many patient studies have also indicated that overexpression of Survivin correlates with increased tumour invasion and metastasis [66, 67], thereby implicating a wider role for Survivin beyond regulating apoptosis and cell division of cancer cells.

### Survivin and cancer stem cells

Existence of cancer stem cells has been proposed as a primary reason for genesis of cancer and disease relapse. It is hypothesized that tumours are maintained by a subset of tumour stem cells that possess the self-renewal capacity similar to stem cells. Role of Survivin has been shown in regulation of adult stem cell physiology such as in haematopoietic stem cells, neuronal stem cells or intestinal stem cells [68–70]. Survivin is also important for embryonic stem cell and totipotent stem cell function [71]. Since normal stem cells and cancer stem cells share common features, it is plausible to assume that like normal stem cells, Survivin expression on cancer stem cells may also be involved in regulating cancer stem cell behaviour.

An investigation of co-expression of Survivin and stem cell specific proteins in oesophageal squamous cell carcinoma (ESCC) patients revealed that patients exhibiting high expression of both a stem cell specific protein Oct-4 and Survivin showed worst prognosis. Survivin expression correlated with Oct-4 expression in ESCC cells suggesting a regulation/interaction between Survivin and Oct-4 [72]. Molecular mechanisms underlying the interaction/regulation between Survivin and stem cell specific proteins are still not properly understood. Role of Survivin is also implicated in specifically regulating genes involved in leukemia cancer stem cell (LCSC) fate and not normal hematopoietic stem cell (HSC). This difference in Survivin signalling in LCSC vs HSC opens new avenues for specific therapeutic targeting and elimination of cancer stem cells [73].

### Survivin as therapeutic target in cancer

Survivin commands a central position among IAPs as being both a regulator of cell division and apoptosis. However, normal differentiated cells have very low or no expression of Survivin. Does low or no expression of Survivin on normal cells have any correlation with cellular functions or less cell division/apoptosis in normal cells? Survivin knock-out mice exhibit embryonic lethality [74], loss of self-renewing bone marrow progenitor cells and bone marrow ablation [75]. Conditional deletion of Survivin in thymus leads to a developmental block in thymocytes at double negative stage and presence of immature T cells in periphery [76]. Survivin also emerges as a key regulator of clonal expansion of Teff cells [77], proliferation of early B cell progenitors and activated mature B cells [78], erythroid [79] and megakaryocyte differentiation [80]. Survivin appears to be an intriguing protein regulating functions of various immune cells, hence therapeutic approaches aimed at targeting Survivin need to be carefully evaluated.

#### Therapeutic approaches targeting Survivin

Due to important role of Survivin in tumor cell division, apoptosis, chemo resistance and cancer stem cell survival; therapeutic blockade of Survivin in tumor cells may possibly yield cumulative benefits. Various strategies have been envisaged to block the expression or function of Survivin in tumour cells (i) immunotherapeutic approaches to induce immune response against Survivin, (ii) small molecule inhibitors/antagonists to block function of Survivin, (iii) nucleic acid based approaches which interfere with Survivin gene expression or (iv) gene ablation of Survivin to regulate cell cycle and apoptosis.

#### Survivin based Immunotherapeutic approaches

Manipulation of host immune system to produce an anti-tumour response is known as immunotherapy. This concept was first pioneered by William Coley (1891) who used extracts of *S. pyogenes* and heat-killed *Bacillus prodigious* (now reclassified as *Serratia marcecsens*) bacteria to treat sarcomas [81]. Coley’s experiments established that immune stimulatory components present in “Coley’s toxins” boosted anti-tumor immune response and induced disease remission in cancer. Coley’s experimental results were widely criticized and concept of immunotherapy was totally dismissed as an effective strategy to control cancer. Alternative treatment modalities in cancer like chemotherapy and radiotherapy which had been well developed by that time presented with drawbacks of inducing disease resurgence and toxicity. Simultaneously, with better understanding of principles of immunology, it was apparent that immune system had a crucial role in controlling certain types of cancer. As various principles of immunology were being unravelled, it became clear that immunotherapy is a better therapeutic option as compared to chemotherapy or radiotherapy because it is specific for tumor cells and is devoid of side effects.

**Fig. 3:**
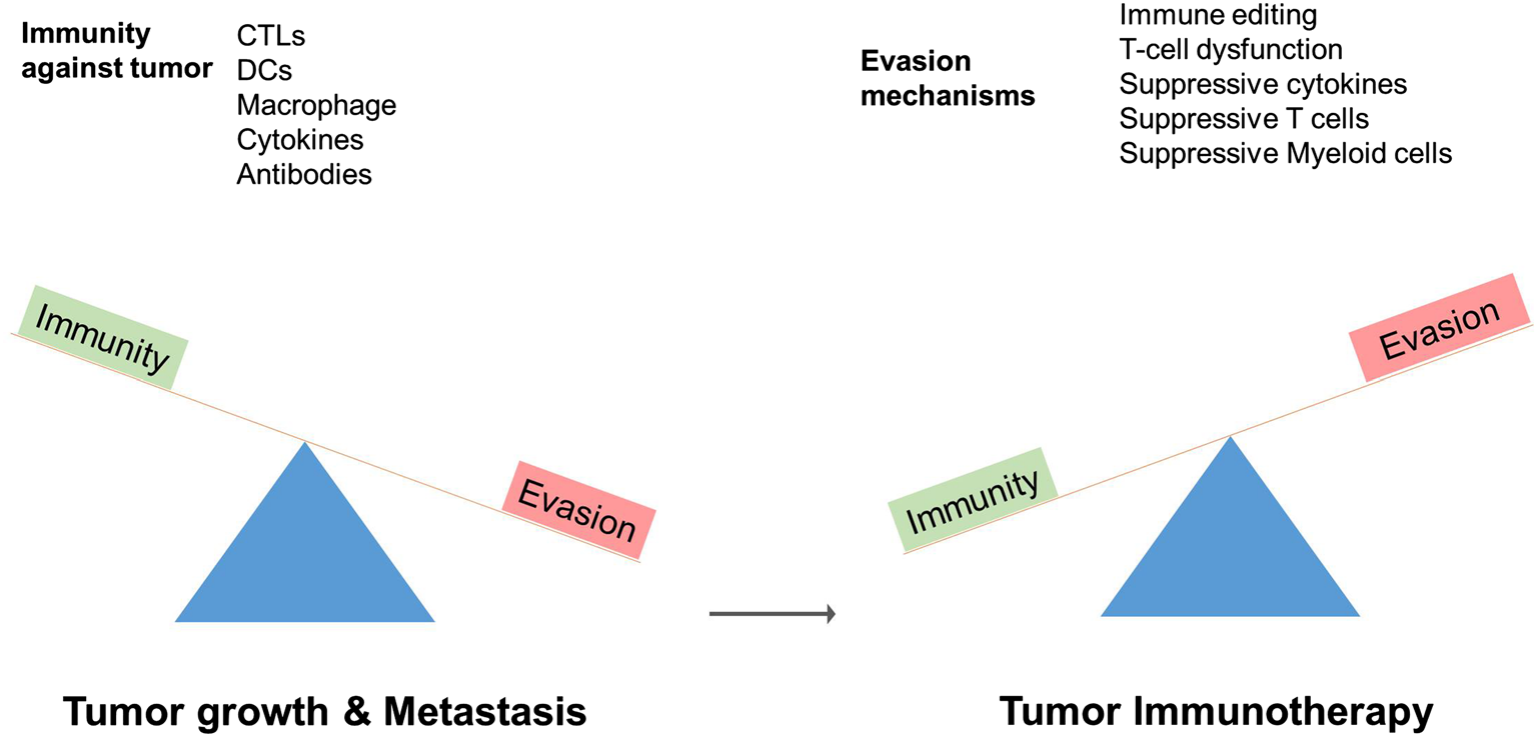
Immunotherapy in cancer. The self-defence of tumors overweighs the anti-tumor immunity leading to clinical manifestations of cancer. The various immunotherapeutic approaches are adopted to reverse this imbalance

Immunotherapy eliminates tumor cells by initiating/boosting anti-tumor immune response or reversing inhibitory immune signalling. Active immunotherapy involves development of a targeted anti-tumor immune response in the host using specific cancer antigens or ex vivo expansion of effector cells and infusion back into the patient. Passive immunotherapy covers transfer of preformed molecules such as therapeutic antibodies or antigen specific T cells which can eliminate or suppress tumor. A number of monoclonal antibodies against growth factor receptors or antigenic determinants on cancer cells have been successfully translated for therapeutic usage in various cancers. Recent success of antibodies targeting inhibitory immune checkpoints such as CTLA-4 and PD-1 has again shifted the focus on inhibitory molecules in immune cell signalling for a beneficial anti-tumor response [82]. Although, diverse approaches for therapeutic cancer vaccines have shown good immunogenicity in clinical trials yet clinical translation of antigen specific cancer vaccines has not been as successful as passive immunotherapy. The rationale for cancer vaccine is to target a specific tumor antigen as vaccine candidate, induce robust antigen presentation mostly through DCs and induction of cytotoxic T cell (CTL) response. Tumor antigens are processed into peptides, loaded onto MHC molecules and MHC-peptide complex is presented by antigen presenting cells (APCs) to T cells for activation. Induction of effector T cell response is sequential; antigen specific T cells are first primed in the secondary lymphoid organs through the interaction with APC. APCs particularly dendritic cells (DC) sample antigens from tumor cells and present antigens to CD4+ T cells via the MHC class-II pathway or to CD8+ T-cells via cross presentation or cross priming [83, 84]. This antigen recognition in association with MHC is insufficient to effectively activate T cells; APC provide additional co-signals that regulate the breadth of T cell activation. These multiple co-signals can be induced by stimulatory (CD80/CD86:CD28) and inhibitory molecules also known as “Immune checkpoints” [85, 86]. Antigen specific T cells once primed by APCs will scan for cognate MHC-peptide on target tumor cells and execute lysis of target cells. Immunotherapy using specific cancer antigens serves to expand the population of antigen specific T cells so that immune escape of tumor is prevented (Fig. 3). The vaccine strategies employ either peptides or whole recombinant protein in adjuvant, recombinant viruses encoding the antigen of interest or other recombinant microorganisms, DNA vaccines, cytokine or co-stimulation enhanced vaccines, killed tumor cells, or DCs pulsed with protein-or peptide. Incorporating a strong adjuvant enhances tumor antigen presentation and activation of immune effector mechanisms leading to induction of a robust CTL response against tumor cells.

An ideal cancer vaccine should use an antigen which is specific and overexpressed on tumor cells. Survivin has emerged as an ideal antigen for cancer immunotherapy because of tumour specific expression and critical role in regulating cell division and apoptosis of tumour cells. [87–89]. Human transcriptome analysis reveals that Survivin is the fourth most highly expressed transcript in human cancer cells when compared to normal cells [90]. Overexpression of Survivin has been associated with an unfavourable prognosis in colorectal and bladder cancers and neuroblastoma [91, 92], melanoma and non-melanoma skin cancers [93, 94]. Survivin overexpression also induces an increased chemotherapeutic drug resistance as discussed earlier in the review. Survivin expression confers a survival advantage on tumour cells and can be a universal therapeutic and diagnostic target for all tumours.

Survivin protein has been shown to induce CTL response in vitro when processed and presented by dendritic cells [95] and Survivin derived peptides prime CTLs in vivo in murine model of melanoma [96]. Generation of Survivin specific CTLs that can lyse HLA matched tumour target cells has been demonstrated in cancer patients [97]. Presence of spontaneous CTL response against MHC-I restricted peptide antigens derived from Survivin in patients suffering from melanoma, breast cancer and leukemia strongly indicates that CD8 T cell restricted epitopes from Survivin are of substantial immunotherapeutic value [98–100]. Induction of effective CTL response was also coupled with homing of Survivin specific CTLs to the site of tumour in situ in melanoma and breast cancer [101] representing a complete synchronization of both local and systemic immune response when using Survivin as a vaccine candidate.

Vaccine approaches such as dendritic cell based (DC) vaccines, DNA vaccines [102], peptide vaccines or VLP based vaccine for Survivin have also been evaluated in preclinical or clinical studies. A DC vaccine trial conducted on non-small cell lung carcinoma (NSCLC) patients who received autologous DCs pulsed with apoptotic bodies of NSCLC showed immunological responses in 4/7 stage III unresectable, and 6/7 stage I/II surgically resected patients [103]. Another vaccine for HLA-A24 positive patients was developed by immunizing HLA-A24 restricted Survivin peptide 2B80–88 which induced peptide specific CTL response in urothelial [104], oral [105], colorectal [106] and breast cancer [107] patients with no adverse effects. In another study, same peptide was combined with IFN alpha and immunised in urothelial cancer patients but had no better results as compared to peptide alone [108]. The peptide alone had low immunogenicity and the investigators concluded that combining the peptide with adjuvants such as heat shock proteins, cytokines etc. may boost immune response to the peptide [107]. A combination of HLA restricted Survivin peptides has also been tested in a phase II trial on metastatic melanoma patients who showed prolonged survival post treatment [109]. A multi-epitope vaccine approach using cocktail of five Survivin peptides: EMD640744 and Montanide(^®^) ISA 51 VG as an adjuvant resulted in anti-Survivin specific T cell responses in patients with solid cancers [110]. A liposome based formulation of Survivin known as DPX-Survivac has also shown to induce Survivin specific immune response in patients with ovarian cancer [111].

Although CTL responses are critical for elimination of tumour cells, role of CD4 T cells and antibodies is also important in modulating anti-tumour immune responses [112]. A number of studies have also shown the important role of CD4 T cells acting as crucial helper cells to boost CTL function and enhance anti-Survivin immune response [113, 114]. Presence of HLA-class II epitopes to stimulate T helper cell response has been demonstrated in Survivin peptides [115, 116]. Sharma et al. have demonstrated an indispensable role of CD4 T cells in sustaining primary and memory anti-tumour immune response in a vaccine formulation containing Survivin evaluated in a transplantable murine model [117]. Importantly, CD4 T cells were crucial in the priming phase of CTLs but not during the effector phase of CTL response. In another important study by Hoffmann et al. recombinant fowl pox virus encoding Survivin derived class I (H1vax1) and class II (H1vax2) derived peptides were designed and evaluated for preclinical efficacy in co-culture experiments using dendritic cells and T cells isolated from healthy donors. These vaccine constructs showed activation of both antigen specific CD4 and CD8 T cells and cytotoxic activity against Survivin-overexpressing mesothelioma cancer cells [118]. Table 1 summarizes the key conclusions obtained from various Survivin vaccines evaluated in preclinical studies or clinical trials. Preclinical studies with Survivin as vaccine candidate indicate induction of an effective anti-tumor response in animal models of solid or haematological malignancies and support the notion that Survivin is immunogenic in humans. However, the immunogenicity may be low enough to provide protection against tumor cells, therefore antigen delivery and role of adjuvants is important to enhance immunogenicity of this protein Furthermore, both CD4 and CD8 restricted epitopes from Survivin protein are important for induction of effective anti-tumour immune response.

**Table 1:** Various vaccine strategies targeting Survivin as vaccine antigen

Vaccine design	Key conclusions	References
Dendritic cell based adenoviral vector expressing full length human mutant Survivin	Significant antitumor effect against three different tumors EL-4 lymphoma, MC-38 carcinoma, and MethA sarcoma. Only partial protection against established tumours observed	[125]
Recombinant fowl pox virus encoding human Survivin	Anti-tumour effect in mouse model of mesothelioma, improved T cell response against tumour cells	[126]
Recombinant mouse Survivin and 4IBBL as adjuvant	Prime-boost vaccination strategy was effective in eradicating 3LL lung carcinoma in 100 % of mice	[127, 128]
Recombinant modified vaccinia Ankara (MVA) expressing full length mouse Survivin	Significant tumor regression and prolonged survival in murine model of pancreatic cancer	[129]
DNA vaccine encoding chemokine CCL21 and Survivin	Oral delivery of this vaccine using attenuated *S. tymphimurium* elicited activation of DCS and effective anti-Survivin CD8 T cells response in a murine model of pulmonary metastasis	[130]
HLA restricted Survivin peptide 2B 80–88	Phase I clinical trial in advanced urothelial patients, showed good CTL response and no adverse effects	[104]
Survivin 2B 80–88 and type I interferon	Clinical improvement in colon cancer patients	[131]
Survivin 2B peptide based vaccination	Clinical improvement in colorectal cancer/breast cancer	[107]
DPX-Survivac using HLA-I restricted Survivin epitopes	Effective anti-Survivin CTL response in ovarian cancer Vaccine induced T cell activation against Survivin antigen	[111, 132]
Autologous dendritic cells pulsed with p53, Survivin and telomerase-derived peptides in combination with low-dose interleukin (IL)-2 and interferon (IFN)-alpha2b in phase I trial	Treatment-associated stable disease (SD) was observed in 24 % of malignant melanoma patients. SD correlated with prolonged survival suggesting a clinical benefit	[133]
EMD640744: a cocktail of five HLA class I-binding Survivin peptides in Montanide (^®^) ISA 51 VG in phase I trial	T-cell responses against Survivin peptides in the majority of patients with solid cancers	[110]

We have also evaluated full length recombinant Survivin protein as a vaccine candidate with *Mycobacterium indicus pranii* (MIP) as an adjuvant in 4T-1 mouse model of breast cancer. MIP has been shown to act as an excellent immune-modulator in animal models for psoriasis, lung cancer and exercises anti-tumor action against Sp2/0 (myeloma) and EL4 (thymoma) in mice models [119]. Subcutaneous administration of MIP-Survivin generated a tumour protective response and tumour regression in 4T-1 model of breast cancer in a dose dependent manner. MIP as an adjuvant was able to induce a good anti-Survivin antibody titre after immunization with recombinant murine Survivin protein. MIP also has been shown to induce DC activation and enhance antigen presentation [120]. Therefore, we propose that combining full length recombinant Survivin with MIP may generate epitopes which can activate both Survivin specific CD8 and CD4 T cells and induce an effective cell mediated immune response. In addition to mediating a direct cell mediated attack of tumour cells, immunization with recombinant Survivin in mice also elicited production of anti-Survivin antibodies. Pre-existing titre of anti Survivin antibodies in Balb/c mice immunized with recombinant Survivin may also have a role in mediating tumour regression in these mice when transplanted with syngeneic 4T-1 cells. Our data suggests that a combination of Survivin and MIP may potentiate anti-tumour immune response (*Garg* et al*. Manuscript under preparation*).

There is limited data on therapeutic efficacy of antibodies against intracellular antigens; however a study from Guo et al. indicates that intracellular antigens can also be targeted by antibodies, thereby widening the scope of targeting both extracellular and intracellular tumour antigens by antibody immunotherapy [121–123]. While direct evidence for role of anti-Survivin antibodies in inducing therapeutic response is lacking, it is possible that induction of anti-Survivin antibodies after antigen immunization may block the overexpression of Survivin on cancer cells or inhibit Survivin expressed on exosomes. Antibodies against Survivin may also promote NK cell lysis of tumor cells. Spontaneous humoral response against Survivin has also been observed in sera of patients of lung, gastrointestinal and colorectal cancer [124]. Importantly, immune response against Survivin (T cell specific or antibodies) was absent in healthy individuals [97, 124] implying that immune response elicited after Survivin vaccination is tumour specific and devoid of autoimmune manifestations. Detailed studies will be required to investigate the effect of Survivin immunization on function of immune effector cells as Survivin has important regulatory role in T cell and B cell effector responses.

#### Small molecule inhibitors of Survivin

Several small molecule inhibitors targeting Survivin have also been evaluated in various in vitro and in vivo studies. Despite the challenges associated with small molecule drug development, these inhibitors are important for understanding Survivin biology and anti-cancer drug development [134, 135]. YM-155 is a novel small molecule which suppresses transactivation of Survivin through direct binding to its promoter [136]. It selectively suppresses expression of Survivin and induces apoptosis in p53-deficient cancer cells in vitro [136].YM155 has also shown to be effective in in vivo models of prostate, pancreatic, and lung cancer [136–138]. Terameprocol (EM-1421), a plant derived small molecule is a novel transcription inhibitor which suppresses Survivin gene expression and induces apoptosis of cancer cells [139]. It is in Phase II safety studies as a vaginal ointment in HPV-linked cervical intraepithelial neoplasia [140]. GDP366 is a novel small molecule compound which potently and selectively inhibited the expression of both Survivin and Op18; an onco-protein [141]. Another small molecule Survivin inhibitor FL118 exhibited superior antitumor efficacy in human tumor xenograft models in comparison to standard anti-cancer drugs [142]. Anti-sense approaches to block Survivin have also been tested. LY2181308, a novel 2′-O-methoxymethyl modified anti-sense oligonucleotide (2-MOE-ASO), is a specific inhibitor of Survivin mRNA and being investigated for efficacy in clinical trials in various groups of cancer patients [143]. Ribozyme mediated approaches have also been evaluated for inhibition of Survivin expression. Down-regulation of human Survivin gene expression and increased apoptosis was achieved by using two hammerhead ribozymes (RZ-1, RZ-2) targeting human Survivin mRNA [144]. Specific Survivin small interfering RNA (siRNA) also inhibited Survivin expression in HL-60 cells and augmented ability of HL-60 cells to overcome drug resistance [145]. siRNA aimed at blocking Survivin-hsp90 connection have also shown anti-cancer effects in androgen independent prostate cancer models [146]. Adenovirus delivered Survivin dominant negative mutants (double point mutant (TC34, 84AA) mediated hepatocellular carcinoma suppression [147]. Another dominant negative mutant (GST-tagged dominant-negative Survivin protein (dNSurR9-C84A) constructed by mutation of Cys84 to Ala in the extreme C-terminal region of the BIR domain of Survivin completely abrogated Survivin’s ability to inhibit apoptosis in cancer cell lines [148]. Almost all small molecule inhibitors of Survivin have been designed based on protein- protein interactions between Survivin and other partner proteins. Since Survivin functions through multiple mechanisms using a variety of partner proteins, blocking one pathway may not entirely lead to reduction of Survivin signalling. Substantial evidence exists in cancer cells for compensatory proliferative mechanisms to override when one signalling pathway is inhibited.

Small molecules are cost effective and have short development time as compared to immunotherapeutic approaches. Therefore, there is enough reasoning to discover and improve existing small molecule inhibitors of Survivin. Immunotherapy approaches, although complex in terms of translational development, are specific and have long lasting effects. However, tumor adaption and immune evasion is one bottleneck to success of immunotherapy strategies. Moreover, either small molecule or immunotherapy targeting Survivin alone has not demonstrated a completely curative response therefore a synergistic combination of small molecules with immunotherapy may be explored to induce and sustain tumor elimination over a course of time.

## Conclusions

**Fig. 4:**
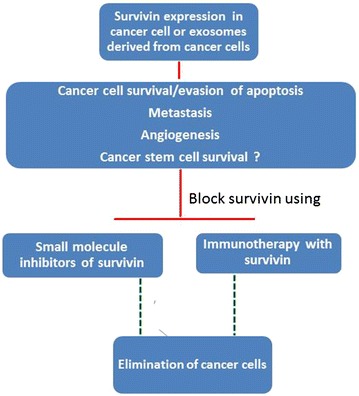
Multiple functions of Survivin. Survivin overexpression suppresses apoptosis or induces abnormal cell division, promotes tumour angiogenesis or metastasis of tumour cells. Survivin may also be expressed extracellularly in form of exosomes and exacerbate disease. Survivin expression may also influence cancer stem cell fate. Therapeutic targeting of Survivin through Survivin inhibitors may serve to block Survivin expression and induce removal of cancer cells. Immunotherapy with Survivin will boost generation of immune response against Survivin by generating Survivin specific CTLs

Survivin has emerged as a unique and nodal molecule mediating diverse roles in cancer cells. However occurrence of different subcellular pools and splice variants of Survivin complicate the biology of this molecule. Occurrence of particular splice variants in various stages of tumor development needs to be thoroughly investigated for therapeutic translation of Survivin blocking approaches. It is also intriguing to note that differential cellular localization of Survivin has different functional outcomes. While its role in cell division and apoptosis has been substantially investigated there is still paucity of data on contribution of Survivin to immune evasion by tumor cells. Circulating tumor cells also express high levels of Survivin and may also be responsible for immune escape of tumor cells. Chronic inflammation and immune evasion may work synergistically to induce Survivin upregulation on tumor cells. Nonetheless, there has been an increasing interest to develop immunotherapeutic approaches targeting Survivin because of its prominent role in cancer development and ability to induce an effective CTL response. Several studies have shown promising results in preclinical evaluation while some have advanced to stage- I or -II clinical trials. The safety and toxicity profile for Survivin has also shown promising outcome. Figure 4 summarizes conceivable benefits of using Survivin inhibitors or immunotherapeutic approaches using Survivin as an antigen. Given the tremendous heterogeneity in human cancers, translational development for Survivin based vaccines or inhibitors of Survivin may even show better results if used in combination with traditional therapies. Combinatorial therapy using Survivin as vaccine and immune checkpoint blockade (PD-1, CTLA-4 etc.) may also be considered for an efficient anti-tumor immune response.
